# Barnyard grasses were processed with rice around 10000 years ago

**DOI:** 10.1038/srep16251

**Published:** 2015-11-05

**Authors:** Xiaoyan Yang, Dorian Q Fuller, Xiujia Huan, Linda Perry, Quan Li, Zhao Li, Jianping Zhang, Zhikun Ma, Yijie Zhuang, Leping Jiang, Yong Ge, Houyuan Lu

**Affiliations:** 1 Key Lab. of Land Surface Pattern and Simulation, Institute of Geographic Sciences and Natural Resources Research, Chinese Academy of Sciences, Beijing 100101, China; 2 Institute of Archaeology, University College London, 31-34 Gordon Square, London WC1H 0PY, U.K.; 3 Institute of Geology and Geophysics, Chinese Academy of Sciences, Beijing 100029, China; 4 University of Chinese Academy of Sciences, Beijing 100049, China; 5 The Foundation for Archaeobotanical Research in Microfossils, PO Box 37, Fairfax, VA, 22038, U.S.A.; 6 Department of Geography and Geoinformation Science, Center for Earth Observing and Space Research, George Mason University, Fairfax, VA, 22030, U.S.A.; 7 Zhejiang Provincial Institute of Cultural Relics and Archaeology, Hangzhou 310014, China; 8 Center for Excellence in Tibetan Plateau Earth Science, Chinese Academy of Sciences, Beijing 100101, China

## Abstract

Rice (*Oryza sativa*) is regarded as the only grass that was selected for cultivation and eventual domestication in the Yangtze basin of China. Although both macro-fossils and micro-fossils of rice have been recovered from the Early Neolithic site of Shangshan, dating to more than 10,000 years before present (BP), we report evidence of phytolith and starch microfossils taken from stone tools, both for grinding and cutting, and cultural layers, that indicating barnyard grass (*Echinochloa* spp.) was a major subsistence resource, alongside smaller quantities of acorn starches (*Lithocarpus/Quercus sensu lato*) and water chestnuts (*Trapa*). This evidence suggests that early managed wetland environments were initially harvested for multiple grain species including barnyard grasses as well as rice, and indicate that the emergence of rice as the favoured cultivated grass and ultimately the key domesticate of the Yangtze basin was a protracted process.

Rice (*Oryza sativa*) is widely regarded as a key domesticated species from the Yangtze River basin in China, and the primary basis of the first agricultural economies in the region^1^,^2^. Recent research has indicated that rice domestication was the result of a protracted evolutionary process in which domesticated rice morphology and economies based on rice cultivation were fully in place only between 6,500 and 6,000 years ago^1^,^3^, but the slow rate of evolutionary change during rice domestication indicates that the beginnings of the process began 3,000 to 4,000 years earlier^4^. One site which has been extensively discussed in this regard, is the archaeological site of Shangshan, which has evidence for rice exploitation dating between 11.0 and 9.0 kyBP^1^,^5^,^6^. However, these earliest phases of the domestication have remained controversial, with debate surrounding whether Shangshan represented pre-domestication cultivation or wild gathering of rice and where on the trajectory towards rice domestication it lies^7^,^8^. Rice remains from the site are limited and lack the evidence for morphological domestication processes available from later sites^3^,^4^. Little attention has been given to other plants that were utilized alongside rice at Shangshan and when rice emerged as the favoured grain species for intensification of use and cultivation. We report evidence from microfossils that indicates that barnyard grass (*Echinochloa* sp.) was a major resource at Shangshan and that rice was just one among a wide spectrum of resources among the hunter-gatherers and early cultivators of the Early Holocene Lower Yangtze. This suggests that rice became the sole favoured grass for domestication after the Shangshan period.

Although preservation of macro-remains of rice and associated flora have been limited, microfossils can provide evidence that complements our picture of plant subsistence, as these may preserve plants and plant parts that were not subjected to the charring conditions that preserve most macro-remains^9^. Therefore comprehensive microfossil studies were undertaken on both lithic implements and sediment samples from previously studied levels of occupation, and we find evidence for the exploitation of nuts, such as acorns (*Lithocarpus/Quercus sensu lato*), and water chestnuts (*Trapa*), as well as extensive evidence for the processing of another wetland grass, barnyard grass (*Echinochloa* spp.), indicating that this wild millet was an important resource harvested and processed alongside rice, and only abandoned in favour of rice at a later stage of the domestication process.

## The Shangshan site and stone tools

**Figure 1:**
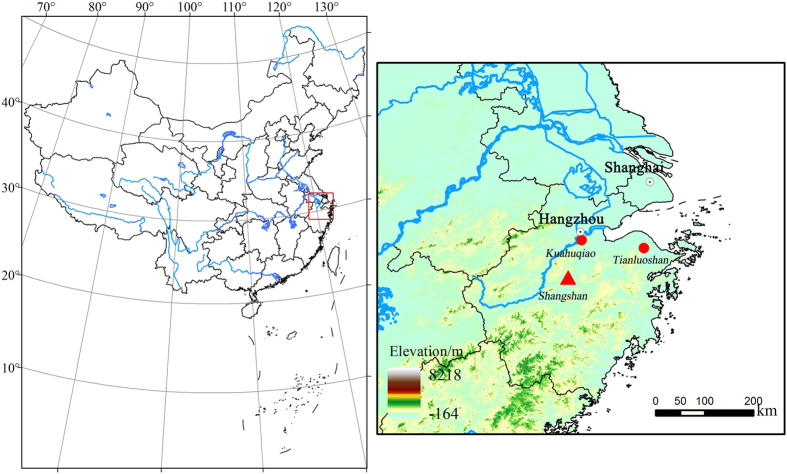
Location of the Shangshan site and other sites mentioned in the text. The shading indicates low-to-high. The firgure1 was generated using DIVA-GIS 7.5 (http://www.diva-gis.org/).

Shangshan is the earliest Neolithic site that has been discovered thus far in the lower Yangtze region. It is situated in a fluvial basin in the upper reaches of the Puyang River, a tributary of the Qiantang River that empties into the East China Sea (Fig. 1). The site is located 50 m above sea level in a hilly area of central Zhejiang Province, just to the south of Hangzhou City (29°27′36″ N, 119°58′25″ E). Three seasons of excavations were completed in 2001, 2004, and 2005–2006 during which more than 1,800 m^2^ of the entire 20,000 m^2^ of the site were exposed. Vestiges of storage pits and house foundations, red pottery tempered with unoxidized plant material, and large numbers of stone tools, including adzes, axes, slabs, and mullers, were unearthed from cultural sediments at Shangshan^10^. AMS radiocarbon dates derived from charcoal samples and pottery temper place the occupations at ca. 11.0–8.6 kyBP, and the site has been divided into three sub-phases, including two phases of Early Shanghan culture up to 9.6 kyBP and the Late Shangshan culture (9.6–8.6 kyBP), which is related to the culture of nearby Kuahuqiao^10^. Despite flotation only a few macro-remains of rice were recovered and most of these came from the upper layers, making inferences about the domestication status of rice or its significance within the overall plant economy difficult to reconstruct^8^,^11^. Additional evidence comes from rice chaff used as ceramic temper and rice phytoliths from cultural deposits^6^.

Nevertheless, the recovery of groundstones that may have served in plant processing, provides another avenues for recovery of information of plants used at the site. Eight lithic tools (Fig. S1) were sampled for the examination of starch grains and phytoliths. In addition three sediment samples collected from three Shangshan occupational phases, and the dust in the storeroom where the artifacts were curated, the top soil at the site, and sediments underlying the cultural deposits were analysed, as these allow for assessment of contamination and validate that recovered starches were associated with the ancient tools (see Materials and Methods).

## Modern Reference Collection

Recovered archaeological starches and phytoliths were compared with a modern reference collection of selected Chinese taxa. The present reference collection includes over 200 species from 20 families, representing those with common economic taxa^12^,^13^,^14^. This reference collection was augmented in particular for the *Echinochloa,* the genus of barnyard and sawa millet. In China, there are eight species in the genus *Echinochloa*^15^, including five true wild taxa and three which evolved after the beginnings of agriculture: two domesticated species (*E. esculenta, E. frumentacea*)^16^ and an obligate rice-mimicking weed, *E. oryzoides*^17^. Wild taxa, especially *E. crus-galli* and *E. colonum* are widespread in wetland habitats throughout tropical and sub-tropical Asia, and often occur as weeds of wet or dry rice fields^18^.

**Figure 2:**
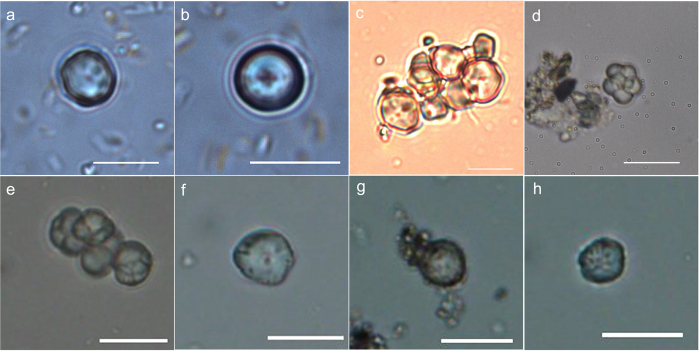
Modern and archaeological starch grains. The upper row is starch grains from modern barnyard grass (**a**) *E. crus-galli*; (**b**) *E. orgzicola*; (**c**) *E. frumentacea*; (**d**) from immature seeds of *E. crus-gulli*; the lower row is archaeological starch grains recovered from residues on the lithic tools excavated from the Shangshan site (Scale bar, 10 μm).

The size of starch grains from barnyard grasses ranges from two or three microns to about 13 microns on the longest axis (Table S1), and the largest starch grains of barnyard grasses can expand to 18 μm in maximum size when processed by grinding in our simulation experiments (Table S1). The unique diagnostic morphological feature of starches from barnyard grasses is a pitted surface that occurs on 20% to 36% of the grains in our modern reference collections (Fig. 2) (Table S1). Surface pits have not been observed in our studies of other modern grass species in the Paniceae.

Barnyard grasses are classified within the tribe Paniceae, and the starch grains from the tribe occur in both polyhedral and spherical forms. Some species, like broomcorn millet (*Panicum miliaceum*) and foxtail millet (*Setarica italica*) produce a predominance of polyhedral starch grains with the occasional spherical form, while other species, including yellow foxtail grass (*Setarica pumila*) and bristly foxtail grass (*Setarica parviflora*), produce exclusively spherical starch grains^12^. Starch grains from barnyard grasses occur in a bimodal distribution with both polyhedral and spherical shapes (Fig. 2a–d), and the relative percentages of the two types differ among the various species (Table S2). All studied species, however, yielded at least 50% spherical granules in their starch assemblages.

**Figure 3:**
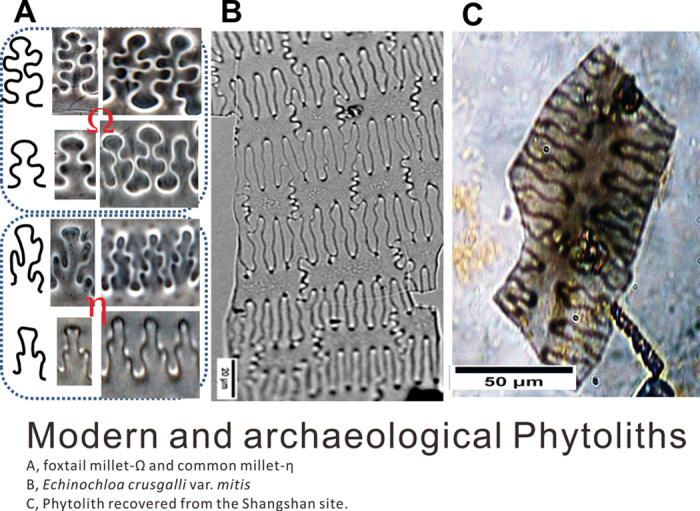
Modern and archaeological phytoliths from barnyard grasses. (**A**) Ω- and η-shaped phytoliths from glumes of foxtail and broomcorn millets, respectively; (**B**) β-shaped phytoliths from glumes of *E. crus-galli* var. *mitis*; (**C**) Phytoliths recovered from the Shangshan site.

Studies of the phytoliths from the husks of barnyard grasses have also defined diagnostic characteristics that allow for the differentiation of these remains from those of other grasses. Phytoliths of epidermal long cells extracted from the lemma-palea of modern barnyard grasses (*Echinochloa crusgalli* var*. mitis*) and related plants have branching patterns that are β-undulated with very shallow sinuous variation (Fig. 3). This characteristic distinguishes barnyard grasses from the Ω-shaped phytoliths in foxtail millet and the η-shaped long cells in broomcorn millet, and our results are consistent with those of previous studies of these grasses^19^,^20^,^21^.

In summary, characteristics of both starch grains and phytoliths are both diagnostic of barnyard grasses and are different from the morphological traits of both closely related grasses and rice. Thus, the recovery of both categories of remains allows for a robust study and clear determination of the presence of these grasses in an archaeobotanical microfossil assemblage.

## Results

**Figure 4:**
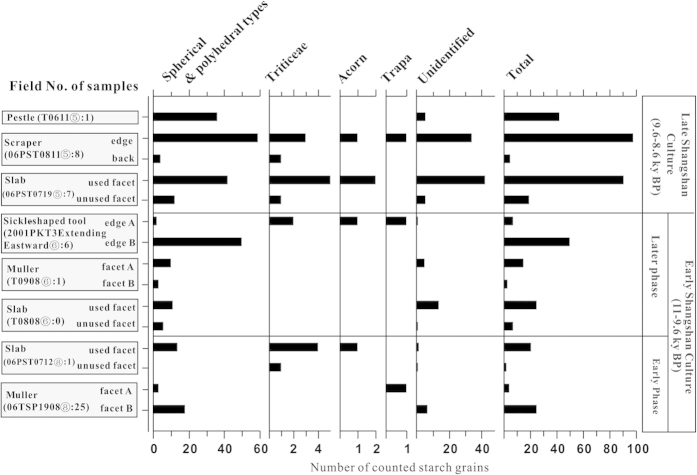
Starch grains counted from surface residues on lithic tools excavated from the Shangshan site.

More than 400 starch grains were recovered from fifteen subsamples of residues extracted from lithic tools (Fig. 4 and Table S3). No starch granules were recovered from the associated sediment samples indicating a likelihood that the ancient starches were associated with tool use. 118 grains were classified as unidentifiable, and this group includes 43 tiny (less than 5 μm), spherical granules which often occur in immature seeds (Fig. S2a), 23 clusters of compound starch grains (Fig. 2h and Fig. S2b), and 52 granules that are damaged to the point that their origin cannot be determined. Though the clusters of compound starch grains are characteristic of those that occur in immature seeds of barnyard grasses (Fig. 2d) and in both mature and immature rice (*Oryza* sp.), similar forms have been noted in many other grasses so these cannot be securely identified^13^,^22^. The single starch grains from rice have polyhedral shape with sharp angles, but they cannot be detected easily and identified securely because of their tiny size^13^. Only a small number of starches from other grass (Triticeae), acorns (*Lithocarpus/Quercus sensu lato*) and water chestnut (*Trapa*) were recovered (Fig. S2c–f). Both acorns and *Trapa* are documented as important food resources alongside cultivated rice at later sites in the region with better preservation of plant macro-remains^23^.

More than 270 starch grains in the assemblage are of a basic polyhedral and spherical morphology (Table S3). Of these, 261 grains fall into the size zone of 5–17 μm, and are characterized by a fissured cavity. Within this subgroup of remains, 54 starch grains, 21% in this assemblage, have pits on their surfaces (Fig. 2f–h). These morphological features are characteristic of barnyard grasses based on the material we have examined. As rice produced starch grains at the smallest end of the starch size range around 5 μm they are much harder to recover and to positively identify. In addition, if rice were boiled as whole grains it would be less likely to be found on groundstone surfaces. Therefore the starch evidence may underrepresent rice, but nevertheless it confirms the importance of *Echinochloa,* another wetland grass with edible grains, which was apparently processed with groudstones.

Phytoliths were studied from both the archaeological layer sediments and the surface residues cleaned off of the grinding tools. 500 phytoliths were counted for each sediment sample from the cultural deposits, and 3,500 phytoliths were recovered from the residues extracted from lithic tools. Of these, 1.5% and 2.0%, respectively, are diagnostic and can be identified to genus or species. The assemblage includes 80 fan-shaped and double-peaked phytoliths that are derived from rice (*Oryza* sp.), respectively from the leaves and husks^9^ (Fig. S2f,g). The other 12 phytoliths are diagnostic of glumes from the Tribe Paniceae (Fig. S2h,i), and this sub-assemblage includes two fragments of phytoliths that bear diagnostic characteristics of barnyard grass (*Echinochloa* spp.) (Fig. 3), which were recovered from the middle level (Later Early Shangshan, >9.6 kyBP). The presence of *Echinochloa* phytoliths in the deposits support the identification based on starch from the groundstone that this taxon was being processed on site. In addition phytoliths affirm the importance of processing rice on site due the presence of both leaf and husk phytoliths.

## Discussion

These data suggest that *Echinochloa* was being harvested and processed alongside rice, and gathered wild foods like nuts and trapa, at Shangshan. *Echinochloa* is well-documented as a group of grasses with food uses. This genus includes two separate domesticated taxa, mawa millet (*E. frumentacea*), domesticated from *E. colonum* in India and found in cultivation eastward to Yunnan^16^, and Japanese barnyard millet (*E. esculenta*), domesticated from *E. crus-galli* before 4.0 kyBP in Japan^24^. In addition, wild *Echinochloa* spp. are reported to be gathered as wild food in Sub-Saharan Africa both in the Early Holocene and modern times^25^,^26^, and as famine foods in India^27^.

*Echinochloa* spp., however, are also common weeds in cultivated rice fields, and are among the most geographically widespread weeds in rice^18^,^28^. *Echinochloa* spp. also co-occur with the wild progenitors of rice, *O. nivara* and *O. rufipogon*^29^. The interpretation of the occurrence of *Echinochloa* grain in archaeological contexts has been varied, with food uses emphasized in Japan and in pre-agricultural Africa^24^,^25^, while research in China and India has usually assigned these the status of weeds^30^,^31^,^32^,^33^. Several factors, however, lead us to believe that *Echinochloa* at Shangshan was being used as a food resource.

First, the remains of barnyard grasses were extracted directly from lithic processing tools, indicating they were processed alongside rice which was clearly being used as a food resource. Second, the ubiquity index of barnyard grass starches on the stone artefacts is 100%. Third, diagnostic barnyard grass starches comprise 21% of the assemblage, with possible *Echinochloa* starches bringing it up to ~50%. These quantitative patterns indicate that it was a routinely processed and more than would be expected if it were a weedy contaminant of rice. We therefore conclude that *Echinochloa* was a significant food resource at Shangshan.

One of the factors favouring use of *Echinochloa* is its high seed productivity. Studies of *Echinochloa* weeds growing with rice report that single plant of *E. colonum* produce 3,000–6,000 seeds, while *E. crus-galli* may have up to 40,000 grains per plant, and as much as 1,000 kg of *Echinochloa* spikelets have been harvested from a single hectare of rice infested with this weed^28^. This is comparable to early rice yields in the Lower Yangtze region which are estimated to have increased from around 1,000 to 2,000 kg/hectare between 0 and 500 AD based on written sources^34^. An estimate of rice yields from the later site of Tianluoshan (6.9–6.6 kyBP) was only slightly lower, 830–950 kg per hectare^32^, This should be considered alongside the changes during the domestication that would have increased the yield of rice plants, implying that earlier crops like those of Shangshan would have been even less productive. The wild progenitor of rice is a perennial, and as such a generally poor grain producer compared to annual relatives or later domesticated rice^1^,^35^. Annuality and improved grain yields is suggested to still be under active selection in the early paddy fields of the Late Majiabang period (~6.0 kyBP)^1^,^36^, implying that Shangshan cultivators had to deal with poor yielding perennial crops. Some domestication traits would have increased the ability of people to harvest a larger proportion of mature filled grains, including dense panicles^34^, and later non-shattering^3^,^37^. These developments would have made harvesting by cutting or uprooting increasingly productive in contrast to forager harvesting methods such as paddle and basket, which is more probable for early crops like those of Shangshan. Basket harvesting would have equally acquired *Echinochloa* or other grasses that grew with the rice, whereas cutting or uprooting could select only for rice plants. Some uprooting or basal cutting has been inferred for Tianluoshan^31^, and the advent of such practices may reflect a choice to focus on rice rather than *Echinochloa* in periods after the Shangshan culture. The archaeobotanical data from Tianluoshan, the only nearby site with large quantitatively studied archaeobotanical macro-remains, also included *Echinochloa* grains (charred) and chaff (waterlogged), but in very low frequencies (more than 1000 times less frequent than rice remains)^31^. This indicates that *Echinochloa* was no longer a significant food resource after 7 kyBP, and may be then have come to be a weed of cultivated rice.

## Conclusion

Microfossil analysis from stone tools and sediments at the Shangshan site produced substantial quantities of starch grains, and phytoliths, of barnyard grasses (*Echinochloa* sp.) from all phases of occupation (11.0–8.6 kyBP). These data indicate that wild *Echinochloa* millets were gathered and processed on groundstone, alongside wild nuts. Rice was also present in phytoliths indicating that it was one among the suite of plants processed on site. While others have discussed the importance of nuts to the earliest rice cultivators^3^,^38^, we have the first hard evidence for use other wild grasses, especially *Echinochloa*, which can be expected in wetland habitats like wild rice. This supports the suggestion that to understand the emergence of agriculture based on domesticated rice, we need to better understand the range of other resources that were utilized and managed alongside the early stages of rice cultivation^38^. It thus appears that the importance of rice as a crop was the outcome of an extended period of experimentation with a broader resource base. One of the earliest transitions may have been from harvesting of managed stands of wetland grasses, which included not just rice but other taxa like *Echinochloa*, towards harvesting and management methods that favoured rice. Through this process rice gradually evolved into a better yielding, domesticated crop, and *Echinochloa* spp. became adapted to cultivation as among the most persistent weeds of cultivated rice.

## Materials and Methods

### Starch grain analysis

The eight grinding stone tools from the Shangshan were sampled at the storage rooms of Pujiang Museum, Zhejiang Province. Two tools from the early phase of Early Shangshan Cultural occupation, three from later phase of Early Shangshan Cultural layer and three from Late Shangshan Culture (Fig. S1). Tools selected for sampling were initially cleaned by brush to remove adhering dust from storage and then washed clean with ultra-pure water. Cavities on the surface of the tools were targeted for residue removal. We applied ultra-pure water to areas of interest and left these to hydrate. The wetted area was agitated with a metal pin to dislodge the sediment within the cavities. Finally a sample of this material was removed with a micropipette and transferred to a clean, new, snap-cap vial for storage. For further the details of the procedure please refer^14^.

These samples were processed in the laboratory at the Institute of geographic Sciences and Natural Resources Research, Beijing. Heavy liquid solutions of CsCl at densities 1.8 was used to float out any starch grains. The recovered residue was mounted in 10% glycerine and 90% water on a slide and examined with both white and cross-polarized light at a magnification of 400×. Starch grains were counted, analyzed for morphological features, then recorded and compared with those from the modern reference collections we complied, which include over 200 starch-producing species from more than 20 families that are common in China^12^,^13^,^14^. Starch grain identifications were based upon one-on-one comparisons between ancient starches and those derived from the modern reference collection.

As a control for the presence of starch, three sediment samples were collected from the dust in the storeroom where the artifacts were curated, the surface soil at the sites, the sediments from the underlayer of the cultural deposits from which the artifacts were recovered. When the control samples were found to either lack starch granules completely, or to have a density of starch that was much lower than the residues on the tools, we took this to indicate that the ancient starches were endogenous to residues on the tools and related to past tool use.

### Phytolith analysis

The samples for phytolith analysis include three sedimentary samples collected from three phases of occupation, and 15 samples of surface residues from used and non-used facets of lithic tools. Recovery of residues for analysis followed that of sampling for starch. The samples were treated with standard procedures of phytolith extraction^39^,^40^. Phytolith nomenclature and descriptions were consistent with International Code for Phytolith Nomenclature 1.0^41^ and Piperno^42^.

## Additional Information

**How to cite this article**: Yang, X. *et al.* Barnyard grasses were processed with rice around 10000 years ago. *Sci. Rep.* **5**, 16251; doi: 10.1038/srep16251 (2015).

## Supplementary Material

Supplementary Information
