# Supplementary material for: Barnyard grasses were processed with rice around 10000 years ago
Source: Sci Rep. 2015 Nov 5;5:16251. doi: 10.1038/srep16251 (PMC4633675; doi:10.1038/srep16251)
Supplement: Supplementary Information [file srep16251-s1.doc]

Supplementary Information for

**Barnyard grasses were processed with rice around 10000 years ago**

Xiaoyan Yang, Dorian Q Fuller, Xiujia Huan, Linda Perry, Quan Li, Zhao Li, Jianping Zhang, Zhikun Ma, Yijie Zhuang, Leping Jiang , Yong Ge, Houyuan Lu

# This file includes:

Table S1, S2, S3

Figure S1, S2

**Table S1** Sizes of starch grains from modern barnyard grass (*Echinochloa* spp.)

| **Species** | **Location** | **Range/µm** | **Mean/µm** | **Source** | ***n.*** | **After grinding/µm** |
| --- | --- | --- | --- | --- | --- | --- |
| *E. frumentacea* Link | Shaanxi, China | 3.3-10.0 | 6.4±1.5 | IB | 100 | 5.2-18.7 |
| *E. frumentacea* Link | Germany | 5.0-12.0 | 8.3±1.6 | IB | 50 | 4.1-13.7 |
| *E. esculenta* (A. Braun) H. Scholz ^1)^ | Czech | 4.2-10.0 | 7.2±1.2 | IB | 50 | 4.3-13.5 |
| *E. colona* (L.) Link | Denmark | 3.8-9.7 | 7.2±1.2 | IB | 50 | 4.1-15.0 |
| *E. crus-galli* (L.) P. Beauv.^2)^ | Yunnan, China | 3.6-9.0 | 6.6±1.2 | IB | 50 | 3.6-12.6 |
| *E. crus-galli* (L.) P. Beauv. | Britain | 5.1-8.8 | 7.2±0.9 | IB | 50 | 4.3-13.2 |
| *E. oryzicola* Vansinger,^3)^ | Heibei, China | 5.7-12.8 | 9.1±1.8 | IB | 50 | 4.2-14.7 |
| *E. crus-galli* (L.) P. Beauv. | China | - | - | IGG |  | Immature |

Notes: IB, Institute of Botany, Chinese Academy of Sciences; IGG, Institute of Geology and Geophysics, Chinese Academy of Sciences. Nomenclature is based on: [www.theplantlist.org](http://www.theplantlist.org/) (accessed 28/2/2015). 1. *E. esculenta* is the accepted name for the synonym *E. utilis* Ohwi & Yabuno. 2. This material was labelled as *E. caudata* Roschev., which is regarded as a synonym for *E. crus-galli.* 3. *E. oryzicola* Vansinger is often regarded as a synonym for *E. oryzoides* (Ard.) Fritsch, but *E. oryzicola* has been retained due to the differences on chromosome number reported by Yabuno (ref. 17).

**Table S2** Percentage of starch grains of different morphologies from barnyard grass (*Echinochloa* spp.)

| **Species** | **Location** | **Polyhedral(%)** | **Spherical (%)** | **Pits on the surface**  **(%)** |
| --- | --- | --- | --- | --- |
| *E. crus-galli* (L.) P. Beauv. | Yunnan, China | 52 | 48 | 36 |
| *E. frumentacea* Link | Shaanxi, China | 28 | 72 | 20 |
| *E. frumentacea* Link | Germany | 38 | 62 | 22 |
| *E. colona* (L.) Link | Denmark | 10 | 90 | 32 |
| *E. esculenta* (A. Braun) H. Scholz | Czech | 18 | 72 | 28 |

**Table S3** Counts of starch grain types from lithic tools and sediment control samples

| **Samples** | **Field No.** | **Subsamples** | **S&P*** | **Triticeae** | **Acorn** | **Trapa** | **UD(T)** | **Total** |
| --- | --- | --- | --- | --- | --- | --- | --- | --- |
| Surface sediment | SS1 | | 0 | 0 | 0 | 0 | 0 | **0** |
| Control dust | MD1 | | 0 | 0 | 0 | 0 | 0 | **0** |
| Underlayer sediment | SS2 | | 0 | 0 | 0 | 0 | 0 | **0** |
| **Early Phase of Early Shangshan Cultural (older than 9.6ka BP)** | | | | | | | | |
| Slab | 06PST0712:1 | unused facet | 0 | 1 | 0 | 0 | 1 | 2 |
|  |  | used facet | 14 | 4 | 1 | 0 | 2(1) | 21 |
|  | **Total** | | **14** | **5** | **1** | **0** | **3(1)** | **23** |
| Muller | 06TSP1908:25 | facet A | 3 | 0 | 0 | 1 | 0 | 4 |
|  |  | facet B | 18 | 0 | 0 | 0 | 7 | 25 |
|  | **Total** | | **21** | **0** | **0** | **1** | **7** | **29** |
|  | **Grand total** | | **35** | **5** | **1** | **1** | **10(1)** | **52** |
| **Later phase of Early Shangshan Cultural (9.6-8.6ka BP)** | | | | | | | | |
| Sickle-shaped tool | 2001PKT3 Extending Eastward:6 | edge A | 2 | 2 | 1 | 1 | 1 | 7 |
|  |  | edge B | >50 | 0 | 0 | 0 | 0 | >50 |
|  | **Total** | | **>52** | **2** | **1** | **1** | **1** | **>57** |
| Muller | T0908:1 | facet A | 10 | 0 | 0 | 0 | 5 | 15 |
|  |  | facet B | 3 | 0 | 0 | 0 | 0 | 3 |
|  | **Total** | | **13** | **0** | **0** | **0** | **5** | **18** |
| Slab | T0808⑥:0 | unused facet | 6 | 0 | 0 | 0 | 1 | 7 |
|  |  | used facet | 11 | 0 | 0 | 0 | 14(4) | 25 |
|  | **Total** | | **17** | **0** | **0** | **0** | **15(4)** | **32** |
|  | **Grand Total** | | **>82** | **2** | **1** | **1** | **21(4)** | **>107** |
| **Late Shangshan Culture (8.6-8.0ka BP)** | | | | | | | | |
| Pestle | T0611⑤:1 | | **36** | **0** | **0** | **0** | **6(2)** | **42** |
| Scraper | 06PST0811⑤:8 | edge | 59 | 3 | 1 | 1 | 34(12) | 98 |
|  |  | back | 4 | 1 | 0 | 0 | 0 | 5 |
|  | **Total** | | **63** | **4** | **1** | **1** | **34(12)** | **103** |
| Slab | 06PST0719⑤:7 | unused facet | 12 | 1 | 0 | 0 | 6 (4) | 19 |
|  |  | used facet | 42 | 5 | 2 | 0 | 42(20) | 91 |
|  | **Total** | | **54** | **6** | **2** | **0** | **48(24)** | **110** |
|  | **Grand total** | | **153** | **10** | **3** | **1** | **88(38)** | **255** |

* S & P indicates spherical and polyhedral starch grains; T: small spherical starch grains similar to transitionary starch grains; UD: undiagnosed starch grains

**Figure S1**


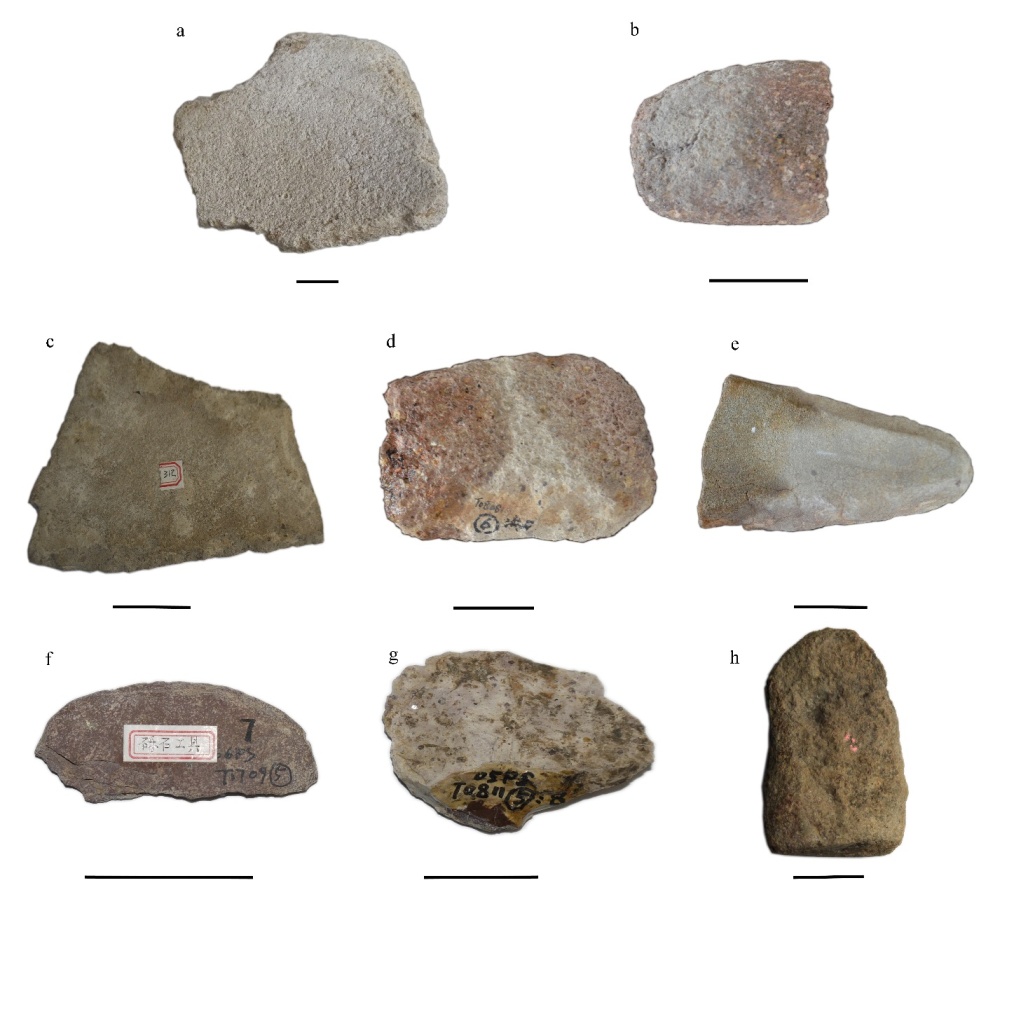


**Figure S1** Lithic tools studied. They were recovered from the early phase of Early Shangshan Culture, later phase of Early Shangshan Culture and Late Shangshan Culture horizons. Discovery contexts and item numbers of the lithic tools from the upper row to the lower row: a, 06PST0712⑧:1; b, 06TSP1908⑧:25; c, 2001PKT3Extending Eastward⑥:6; d, T0808⑥:0; e, T0908⑥:1; f, 06PST0719⑤:7; g, 06PST0811⑤:8; h, T0611⑤:1. Scale bar, 5cm.

**Figure S2**


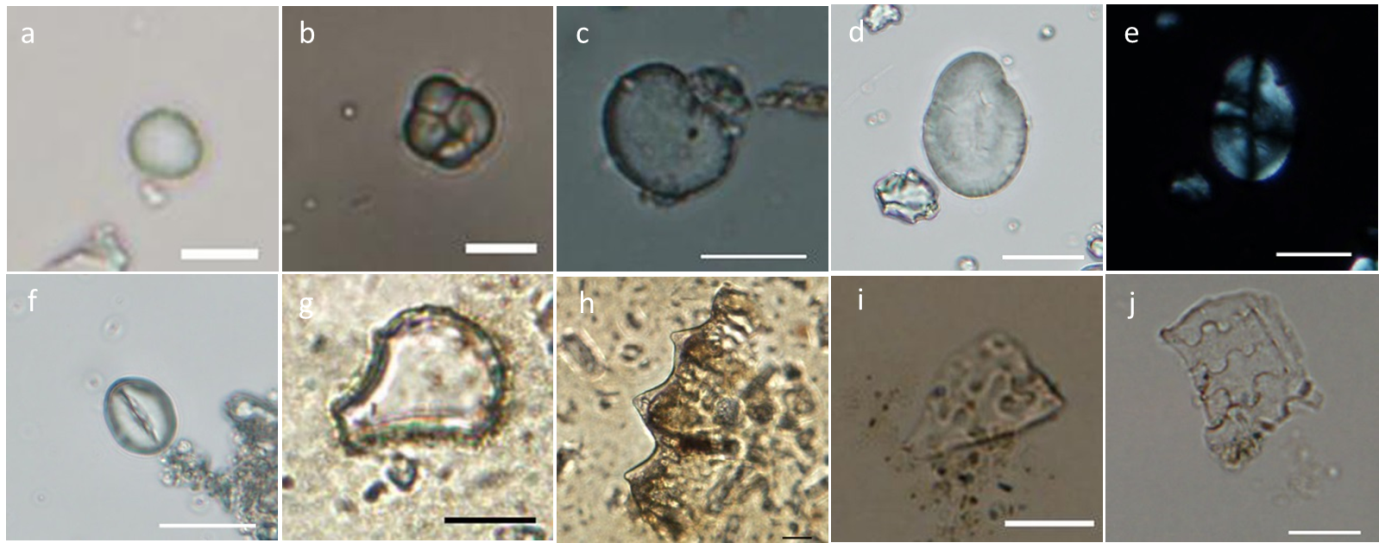


**Figure S2** Archaeological starch grains and phytoliths from the Shangshan site. a, small, spherical starch grain from residues on lithic tools, unidentifiable; b, compound starch grains from residues on lithic tools; c, starch grains from grass Triticeae; d-e, starch grains from water chestnuts (*Trapa*) under bright and polarized light, respectively; f, starch grains from acorns (*Quercus*); g, fan-shaped rice phytoliths from residues and sediments; h, double-peak rice phytoliths from residues and sediments; i-j, phytoliths from glumes of the Paniceae (Scale bar, a-b, 10μm; c-j, 20μm)
